# Application of machine learning algorithm incorporating dietary intake in prediction of gestational diabetes mellitus

**DOI:** 10.1530/EC-24-0169

**Published:** 2024-11-21

**Authors:** Tianze Ding, Peijie Liu, Jie Jia, Hui Wu, Jie Zhu, Kefeng Yang

**Affiliations:** 1 Department of Clinical Nutrition, Xin Hua Hospital Affiliated to School of Medicine, Shanghai Jiao Tong University, Shanghai, China; 2 Department of Clinical Nutrition, College of Heath Science and Technology, School of Medicine, Shanghai Jiao Tong University, Shanghai, China; 3 Department of Nutrition, Seventh People’s Hospital of Shanghai University of Traditional Chinese Medicine, Shanghai, China; 4 Nutrition and Foods Program, School of Family and Consumer Sciences, Texas State University, San Marcos, Texas, USA

**Keywords:** feature selection, gestational diabetes mellitus, machine learning, prediction model

## Abstract

**Introduction:**

Gestational diabetes mellitus (GDM) significantly affects pregnancy outcomes. Therefore, it is crucial to develop prediction models since they can guide timely interventions to reduce the incidence of GDM and its associated adverse effects.

**Methods:**

A total of 554 pregnant women were selected and their sociodemographic characteristics, clinical data and dietary data were collected. Dietary data were investigated by a validated semi-quantitative food frequency questionnaire (FFQ). We applied random forest mean decrease impurity for feature selection and the models are built using logistic regression, XGBoost, and LightGBM algorithms. The prediction performance of different models was compared by accuracy, sensitivity, specificity, area under curve (AUC) and Hosmer–Lemeshow test.

**Results:**

Blood glucose, age, pre-pregnancy body mass index (BMI), triglycerides and high-density lipoprotein cholesterol (HDL) were the top five features according to the feature selection. Among the three algorithms, XGBoost performed best with an AUC of 0.788, LightGBM came second (AUC = 0.749), and logistic regression performed the worst (AUC = 0.712). In addition, XGBoost and LightGBM both achieved a fairly good performance when dietary information was included, surpassing their performance on the non-dietary dataset (0.788 vs 0.718 in XGBoost; 0.749 vs 0.726 in LightGBM).

**Conclusion:**

XGBoost and LightGBM algorithms outperform logistic regression in predicting GDM among Chinese pregnant women. In addition, dietary data may have a positive effect on improving model performance, which deserves more in-depth investigation with larger sample size.

## Introduction

Gestational diabetes mellitus (GDM) refers to carbohydrate intolerance resulting in hyperglycemia of variable severity with onset or first recognition during pregnancy (1, 2). It has become one of the main reasons that seriously affect the pregnancy outcomes (3, 4, 5). The incidence of GDM in China is about 20% and is increasing year by year (6, 7). Though GDM significantly affects the health of pregnant women and their fetuses, the diagnosis of GDM in China now relies on the 75 g oral glucose tolerance test (OGTT) (8, 9). While OGTT is usually performed between the 24th and 28th weeks of pregnancy, it is obviously too late to prevent the occurrence of GDM. A meta-analysis (10) shows that an early intervention (e.g. 15 weeks) can reduce the incidence of GDM, and the earlier the intervention, the better the effect. Therefore, it is important to screen high-risk individuals for GDM at an earlier stage to protect the health of pregnant women and their fetuses from adverse pregnancy outcomes.

GDM is commonly associated with an array of factors, including obesity, a familial history of diabetes and the presence of polycystic ovary syndrome, among others (11, 12, 13). In recent years, these factors have been considered as the predictive factors in the research of the GDM prediction model. For example, in a cohort study of pregnant women in Tianjin, China, age, waist circumference, hip circumference, alanine aminotransferase (ALT), pre-pregnancy BMI, systolic blood pressure, family history of diabetes mellitus and fasting blood glucose were selected as predictive indexes (14). Researchers in Israel and South Korea have carried out similar studies (15, 16).

However, we believe that there is still room for optimization of current machine learning models related to GDM. As a matter of fact, while diets directly affect many important physical indexes such as BMI, blood pressure, serum cholesterol, etc., dietary data are missing in current studies of GDM prediction. In terms of pathogenesis, most GDM are caused by obesity and chronic insulin resistance (17), and these problems are mostly caused by bad dietary habits. Existing studies have shown that dietary factors such as dietary patterns, unreasonable energy ratio of the three major nutrients and excessive intake of carbohydrates are all risk factors for GDM (18, 19, 20, 21, 22, 23). Given the pathogenesis foundation, this study aims to fill the gap by incorporating dietary data as predictive factors for an improved and integrated predictive model. By analyzing complex dietary patterns in patient data and integrating these patterns with other clinical features, the gestational diabetes prediction models can assist clinicians in providing personalized dietary guidance to pregnant women at high risk of GDM.

## Methods

### Data collection

We recruited participants from the Seventh People’s Hospital Affiliated with Shanghai University of Chinese Medicine in Shanghai, China, between January 2018 and June 2021. Our inclusion criteria for pregnant women were (i) natural conception, (ii) singleton pregnancy and (iii) the ability to read, understand, and sign informed consent. We excluded pregnant women who had been diagnosed with diabetes prior to pregnancy, as well as those with severe heart, brain, liver, or kidney diseases, gestational thyroid disease, mental disorders, placenta previa, any infectious diseases, autoimmune diseases or malignant tumors. Ultimately, a total of 554 pregnant women were enrolled in the study.

We collected clinical data from these pregnant women, focusing particularly on routine blood tests and blood lipid examinations conducted between the 10th and 12th weeks of pregnancy. We also carried out dietary surveys and universal screening for GDM at around the 24th week of pregnancy. According to the diagnostic criteria of GDM in China, which refer to the 75g oral glucose tolerance test (OGTT), 152 pregnant women were diagnosed with GDM (24, 25, 26). In addition, a validated semi-quantitative food frequency questionnaire (FFQ) consisting of 222 food-related items was used to assess participants’ dietary intakes through face-to-face interviews. For each item in the FFQ, three aspects were recorded: whether the item was consumed, the usual frequency of consumption (times per day, week, month or year) and the estimated amount consumed per occasion, expressed in either the local unit ‘liang’ for weight (1 liang = 50 g) or a standard cup for volume (1 cup = 250 mL). All researchers conducting the food survey were dietitians with over 3 years of experience in hospitals and received training before the study. To enhance the accuracy of participants’ food recall, food models and pictures were also used. We estimated participants’ average daily intake of oil, salt and sugar based on their household’s total consumption of these condiments. Finally, we expressed all food data as daily intake per person and calculated individual nutrient intakes using a nutritional dietary management information system developed by Shanghai Zhending Health Technology Co., Ltd (27, 28). We collected 78 features in total, but only 77 were used in the actual modeling, as one feature was excluded due to excessive missing values.

Written informed consent was obtained from all study participants. To protect individuals’ privacy, we replaced sensitive information, particularly names, with codes. During data collection, codes were assigned based on hospital admission time and bed number. For statistical analysis, only these codes were retained, and the names of the pregnant women were excluded. This study was registered at www.chictr.org.cn. (ChiCTR1900027764) and was approved by the Medical Ethics Committee of the Seventh People’s Hospital affiliated with Shanghai University of Traditional Chinese Medicine (2020-7th-HIRB-016).

### Model building

#### Missing values handling

We handled missing values in the dataset by removing features with more than 10% missing data, and excluding them from modeling. For the remaining data, missing values in continuous variables were imputed using mean values, while categorical variables were assigned default values: parity was set to 1, and both education level and family history of diabetes were set to zero. The count of missing values for all features is provided in the Supplementary Table 1 (see section on supplementary materials given at the end of this article).

**Table 1:** Comparison of the sociodemographic characteristics between the control group and the GDM group.

Characteristics	Control (402)	GDM (152)	*P*
Age/year	28.43 ± 4.42	30.79 ± 4.68	<0.001
Height/cm	161.00 ± 4.84	159.85 ± 4.55	0.005
Pre-pregnancy Weight/kg	57.02 ± 10.05	60.83 ± 12.30	0.001
Pre-pregnancy BMI/kg∙m^0-1-2^	21.93 ± 3.44	23.98 ± 6.17	<0.001
Parity/*n* (%)			0.072
Primiparity	209 (52.0)	66 (43.4)	
Multipara	193 (48.0)	86 (56.6)	
Number of pregnancies/*n* (%)			0.017
Primigravida	169 (42.1)	47 (30.9)	
Multigravida	233 (57.9)	105 (69.1)	
Education level/*n* (%)			0.981
Below college	317 (78.9)	120 (78.9)	
College and above	85 (21.1)	32 (21.1)	
Exercise/*n* (%)			0.836
Yes	282 (70.2)	108 (71.1)	
No	120 (29.8)	44 (28.9)	
History of miscarriages/*n* (%)			0.295
Yes	142 (35.4)	61 (40.2)	
No	260 (64.6)	91 (59.8)	
Family history of diabetes/*n* (%)			0.012
Yes	34 (8.5)	24 (15.8)	
No	368 (91.5)	128 (84.2)	
History of diabetes/*n* (%)			<0.001
Yes	3 (0.7)	16 (10.5)	
No	399 (99.3)	136 (89.5)	

GDM, gestational diabetes mellitus.

#### Feature selection

In this study, random forest mean decrease impurity was applied for feature selection.

#### Two datasets

Dietary data are important but not studied in predictive models for GDM. Therefore, in this study, we created two datasets containing different features. The features in one dataset included sociodemographic and clinical data; the other dataset included sociodemographic data, clinical data and dietary data. We compared the predictive performance of the models constructed with these two datasets separately. In this way, we can find out whether dietary data can improve the performance of the models.

#### Model training

Logistic regression, XGBoost and LightGBM algorithms were utilized. To maximize the use of the limited data, a five-fold cross-validation was performed to split the training and test sets, and Grid Search was employed to find the optimal parameters.

#### Model evaluation

Accuracy (Ac), sensitivity (Sn), specificity (Sp), areas under the curve (AUC) of receiver operating characteristic (ROC) and Hosmer–Lemeshow test (HL test) were used to evaluate the performance of the models. All the construction and evaluation of the models were conducted in Python (version 3.11.9) using Visual Studio Code (version 1.91.1).

### Statistical analysis

Descriptive analysis was performed to determine the numbers and percentages for sociodemographic data. Group differences in sociodemographic, clinical, and dietary data were analyzed using an independent samples *t*-test, chi-square test, or Mann–Whitney *U* test. All analyses were performed using IBM SPSS Statistics, version 29 and statistical signiﬁcance was deﬁned as *P* < 0.05.

## Results

### Baseline characteristics

A total of 554 pregnant women were included in the final data analysis. The quantitative data were expressed as mean ± s.d., and the qualitative data were expressed as *n* (%). Among the 554 participants, there were 402 pregnant women in the control group and 152 pregnant women in the GDM group. The results showed that there were significant differences in age, height, pre-pregnancy weight, pre-pregnancy BMI, number of pregnancies, family history, and diabetes history between the control group and the GDM group (*P* < 0.001, *P* = 0.005, *P* = 0.001, *P* < 0.001, *P* = 0.017, *P* = 0.012, *P* < 0.001). There were no significant differences in parity, education level, exercise and history of miscarriages between the two groups. The results are detailed in Table 1.

### Feature selection

**Figure 1:**
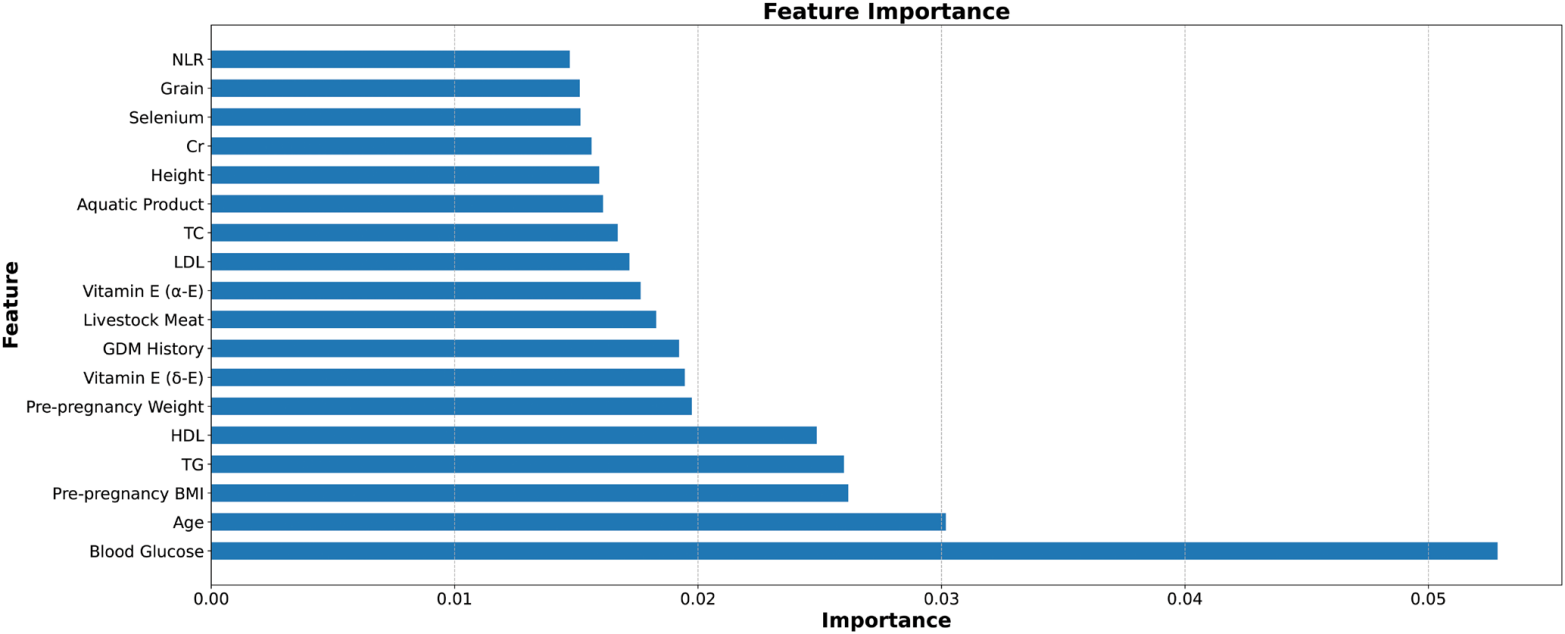
The features selected by random forest mean decrease impurity. Cr, serum creatinine; NLR, neutrophil lymphocyte ratio; TC, total cholesterol; TG, triglyceride.

After cleaning the data and eliminating redundant variables, we extracted a total of 18 out of 77 features by random forest and arranged these features as follows (Fig. 1). Blood glucose, age, pre-pregnancy BMI, triglycerides and HDL were the top five features. In dietary indexes, vitamin E (δ-E), livestock meat, vitamin E (α-E), aquatic products, selenium and grain contributed remarkably to the outcome.

### Model evaluation

#### Discrimination of different models

**Figure 2:**
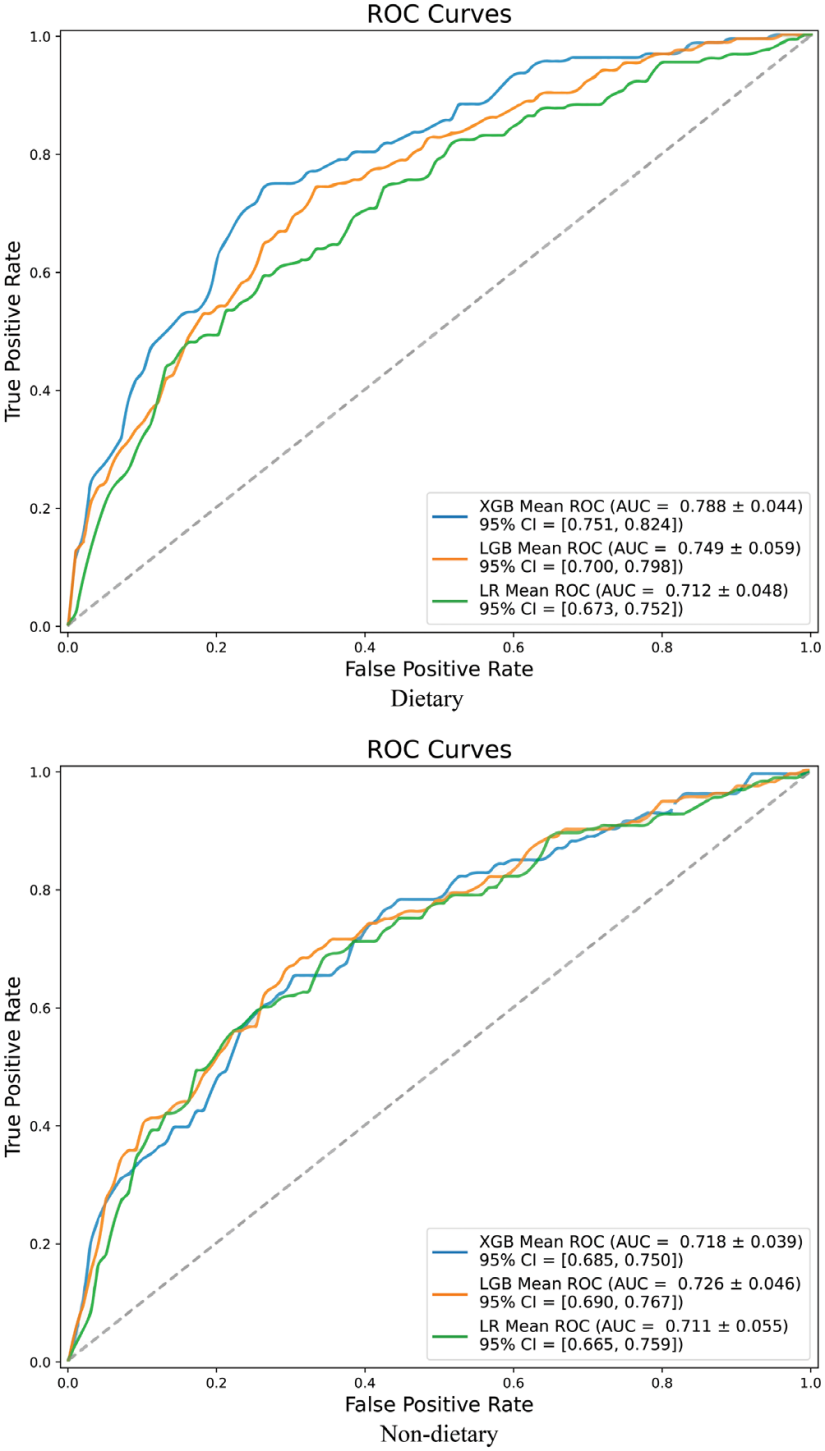
The ROC curves of the three GDM models. LGB, LightGBM; LR, logistic regression; XGB, XGBoost.

The ROC curves and confusion matrixes of the three models and the two datasets are shown in Fig. 2 and Supplementary Figure 1. A table of Ac, Sp, Sn and AUC is also provided (Table 2). The logistic regression (LR) model has the lowest performance (AUC = 0.712) and the conclusions of two datasets are almost the same. Of the other two models, XGBoost model performs better with an AUC of 0.788, while the AUC of LightGBM model is 0.749. Moreover, in both the XGBoost and LightGBM models, dietary dataset outperforms non-dietary dataset (0.788 vs 0.718 in XGBoost; 0.749 vs 0.726 in LightGBM), which indicates that dietary data may improve the prediction accuracy of the models.

**Table 2:** Predictive performance of the three GDM models.

Algorithm	Dataset	Accuracy	Sensitivity	Specificity	AUC (95% CI)
LR	Dietary	0.755	0.270	0.938	0.712 (0.673, 0.752)
	Non-dietary	0.747	0.230	0.943	0.711 (0.665, 0.759)
XGB	Dietary	0.769	0.322	0.938	0.788 (0.751, 0.824)
	Non-dietary	0.744	0.283	0.918	0.718 (0.685, 0.750)
LGB	Dietary	0.747	0.309	0.913	0.749 (0.700, 0.798)
	Non-dietary	0.764	0.362	0.918	0.726 (0.690, 0.767)

LGB, LightGBM; LR, logistic regression; XGB, XGBoost.

#### Calibration of different models

The HL test was used to test the calibration of the LR, XGB and LGB models (Table 3 and Supplementary Figure 2). Most models are well-calibrated since their *P* values are over 0.05. However, the *P*-value of the LGB models with dietary data is below 0.05, which indicates that the model’s calibration is poor.

**Table 3:** The calibration of the three GDM models.

Algorithm	Dataset	HL-chi2	*P*
LR	Dietary	9.47	0.304
	Non-dietary	6.44	0.598
XGB	Dietary	12.06	0.149
	Non-dietary	11.64	0.168
LGB	Dietary	21.66	0.006
	Non-dietary	8.67	0.371

HL, Hosmer–Lemeshow test; LGB, LightGBM; LR, logistic regression; XGB, XGBoost.

## Discussion

Our study used three different machine learning algorithms (logistic regression, XGBoost, LightGBM), two different datasets (dietary dataset and non-dietary dataset) and eighteen different features (sociodemographic data, clinical data, and dietary data) to establish predictive models for GDM. As a result, overall, models with the dietary dataset performed better than models with the non-dietary dataset, and the XGBoost algorithm was the best of the three.

Our results show that incorporating dietary data into the models improves their predictive performance to some extent. As to XGBoost and LightGBM, the AUC values of the models with dietary datasets are better than those of the models with non-dietary datasets (0.788 vs 0.718 in XGBoost; 0.749 vs 0.726 in LightGBM). The reason for this could be that many dietary factors are potentially associated with the development of GDM. A prospective cohort study in Western China (29) found that a high protein-low starch diet was associated with a lower risk for GDM among women who were overweight at pre-pregnancy, which indicated that dietary patterns can affect the incidence of GDM. We also found a significant difference in the energy contribution ratio of the three macronutrients between the GDM group and the control group (Supplementary Table 2). Many other studies (18, 19, 20, 21, 22, 23) also explored the relationship between diet and GDM from the perspective of dietary patterns and nutrients and have obtained conclusions that possess statistical significance. However, in the logistic regression models, the performance of the dietary dataset was nearly the same as that of the non-dietary dataset. This may be due to the fact that logistic regression is better at dealing with linear problems, whereas for datasets with weak linear relationships such as GDM, the performance of the model is poor and unstable.

Among the algorithms, XGBoost (AUC = 0.788 with the dietary dataset) performed the best, followed by LightGBM (AUC = 0.749 with the dietary dataset), while LR had relatively low predictive performance (AUC = 0.712 with the dietary dataset). This could be because, as mentioned in the previous paragraph, our dataset is complex, and logistic regression algorithms are not well-suited for handling non-linear relationships. Consequently, XGBoost and LightGBM perform better in prediction. When we compare the two again, XGBoost performs better and avoids overfitting. In contrast, LightGBM uses a leaf-wise growth strategy, which can result in deeper decision trees and a higher likelihood of overfitting, especially in models with smaller datasets. In our study, LightGBM very likely caused overfitting, which may explain why it underperforms compared to XGBoost. In addition, LightGBM’s advantages over XGBoost include lower memory usage and faster runtime.

When it comes to the calibration of the three models, LR is the best calibrated, this may be because logistic regression models the probability of a binary outcome directly using the logistic function. This means that the output is a probability that can be interpreted as the confidence of the prediction, which aligns naturally with the concept of calibration. But that doesn’t make much sense since it has a poor AUC value. Meanwhile, as mentioned above, the *P* value of the LightGBM model with dietary data is under 0.05, which indicates that although the model was able to distinguish the high-risk status of GDM, the specific risk probabilities provided by the model are not good (30). The reason for the poor calibration of LightGBM is most likely due to the occurrence of overfitting. LightGBM can build very deep decision trees, which can capture complex patterns in the data. However, deeper trees and a larger number of features are likely to make the model overly sensitive to specific details in the training data. This can lead the model to seek out complex relationships between features and the target variable, causing it to learn the noise in the dataset, ultimately resulting in overfitting. In contrast, when the number of features decreases (the non-dietary model), the calibration of LightGBM becomes better. Besides reducing the number of features, another way to address this issue is to increase the sample size. When it comes to XGBoost, the *P*-values for the HL test were all above 0.05, indicating good calibration. In summary, the XGBoost model with the dietary data has the highest AUC value while guaranteeing excellent calibration and is the best model in our study.

The models we built have similar or even higher AUC values compared to other studies that predict gestational diabetes (14, 31, 32, 33, 34). Though these studies also added features that are the main factors of GDM, such as pre-pregnancy BMI, age, history of GDM, etc. (35, 36, 37, 38), none of them takes dietary data into account and has a lower dimension than ours. This is probably the reason why our models have better performance. While at the same time, there are other models that have higher AUC values than ours, mostly higher than 0.80 (15, 39). This may be because they have a larger sample size. In the study conducted in Israel (15), researchers collected data from electronic health records (EHRs) and used the built-in algorithm to predict GDM. As a result, they had an AUC value of 0.85. Due to the convenience of EHRs, they have a sample size of 588,662 pregnant women and their diagnosis of GDM is different from ours, which may also be the reason that their model has a better performance. Meanwhile, Wang *et al.*’s study (39) also obtained an AUC value of more than 0.80. In their study, they used a score-scaled model, logistic regression model, decision tree model, and random forest model to predict GDM. However, in theory, the predictive performance of our XGBoost model and the LightGBM model are no worse, or even better. The reason for this may be that they included some variables in the model that we didn’t have, such as the positivity of thyroid-related antibodies and gestational weight gain (35, 36, 38, 40).

Our research has several strengths and limitations. A key strength is the integration of dietary data, which can enhance the performance of machine-learning models used to predict the onset of GDM. This means that incorporating dietary information can help build more accurate predictive models. However, our study has two primary limitations. First, the small sample size may have contributed to the suboptimal calibration of the LightGBM model. Increasing the sample size could improve the model’s calibration and overall predictive performance. Second, the dietary survey was conducted around the 24th week of pregnancy, later than the clinical data collection. Although the FFQ reflects dietary intake over the past 24 weeks and offers some insights into early pregnancy diet, it weakened the application value of predictive models. The significance of our research lies in the discovery that introducing dietary data is beneficial for predicting GDM. In future studies, an earlier dietary survey time point (such as about 10 weeks of early pregnancy) and a more accurate artificial intelligence dietary survey method can be explored to improve prediction accuracy.

In conclusion, this study established three predictive models of GDM to help clinicians provide intervention and dietary guidance to pregnant women at high risk of GDM. Among them, the model used XGBoost algorithm and dietary dataset performed best. It is a significant finding that dietary data do improve the predictive performance of the models. The feature selection methods and machine learning algorithms used in this paper can also help the following researchers build more accurate predictive models, especially with a larger sample size.

## Supplementary Materials

Supplementary Material

## Declaration of interest

The authors declare that there is no conflict of interest that could be perceived as prejudicing the impartiality of the study reported.

## Funding

National Natural Science Foundation of Chinahttp://dx.doi.org/10.13039/501100001809 (81573140); 2022 Multidisciplinary Internal Research Grant at Texas State Universityhttp://dx.doi.org/10.13039/100008882; THRC/CHERR Faculty Fellowship Funding.

## Ethics approval and consent to participate

This study was registered at www.chictr.org.cn (ChiCTR1900027764) and was approved by the Medical Ethics Committee of the Seventh People’s Hospital affiliated to Shanghai University of Traditional Chinese Medicine (2020-7th-HIRB-016). Written informed consent was obtained from all study participants.

## Availability of data and materials

Although the datasets generated during the study are not publicly available due to limitations of ethical approval, researchers can get them from the corresponding author on reasonable request.

## Authors’ contribution statement

KY, JZ: study design and concept; TD, LP: programming and manuscript drafting; TD: data analysis and result interpretation; JJ, HW, KY: data collection and critical revision of the manuscript; and all authors: read and approved the final manuscript. None of the authors reported a conflict of interest related to the study.

## Acknowledgements

We sincerely thank all the women who participated in this study and their families for their cooperation. We also thank all the students and researchers who participated in the dietary survey. This study could not have been completed without your help.
