# Supplementary material for: Application of machine learning algorithm incorporating dietary intake in prediction of gestational diabetes mellitus
Source: Endocr Connect. 2024 Nov 21;13(12):e240169. doi: 10.1530/EC-24-0169 (PMC11623027; doi:10.1530/EC-24-0169)
Supplement: Supplementary Material [file supplementary_material.pdf]

Supplements

Title: Application of Machine Learning Algorithm Incorporating Dietary Intake in Prediction of Gestational Diabetes Mellitus

First Author: Tianze Ding

eTable 1. The count of missing values for all features

eFigure 1. The confusion matrixes of the three GDM models

eFigure 2. The calibration curve for the test set of the three GDM models

eTable 2. Comparison of Dietary Data and Clinical Data Between the Control Group and the GDM Group

This supplementary material has been provided by the authors to give readers additional information about their work.

eTable 1. The count of missing values for all features

Features	Count of Missing Values (Total = 554)
Energy (Kcal)	3
Protein (g)	3
Fats (g)	3
Carbohydrates (g)	3
Dietary Fiber (g)	3
Calcium (mg)	3
Iron (mg)	3
Zinc (mg)	3
Selenium (μg)	3
Copper (mg)	3
Manganese (mg)	3
Magnesium (mg)	3
Sodium (mg)	3
Potassium (mg)	3
Phosphorus (mg)	3
Retinol (μgRE)	3
Vitamin E (mg)	3
Vitamin B1 (mg)	3
Vitamin B2 (mg)	3
Ascorbic acid (mg)	3
Niacin (mg)	3
Cholesterol (mg)	3
Water (g)	3
Iodine (μg)	3
Vitamin E (α-E) (mg)	3
Vitamin E ((β+γ)-E) (mg)	3
Vitamin E (δ-E) (mg)	3
Grain (g)	3
Legumes (g)	3
Vegetables (g)	3
Fungus and Algae (g)	3
Fruits (g)	3
Poultry (g)	3
Livestock Meat (g)	3
Dairy (g)	3
Eggs (g)	3
Aquatic Product (g)	3
Energy Composition Ratio (Carbohydrates) (%)	3
Energy Composition Ratio (Fats) (%)	3
Energy Composition Ratio (Protein) (%)	3
Energy from carbohydrates (Kcal)	3
Energy from fats (Kcal)	3

Energy from protein (Kcal)	3
high-quality protein	3
composition ratio of high-quality protein	3
low-quality protein	3
composition ratio of low-quality protein	3
animal-derived fats	3
composition ratio of animal-derived fats	3
plant-derived fats	3
composition ratio of plant-derived fats	3
SFA composition ratio	3
MUFA composition ratio	3
PUFA composition ratio	3
Cr (μmol/L)	2
Blood Sugar (mmol/L)	1
WBC (10^9/L)	1
NLR	1
RBC (10^9/L)	1
HB (g/L)	2
HCT (%)	2
PLT (10^9/L)	1
CRP*	438*
TC (mmol/L)	45
TG (mmol/L)	45
HDL (mmol/L)	45
LDL (mmol/L)	45
Age/year	9
Height/cm	12
Pre-pregnancy Weight/kg	12
Pre-pregnancy BMI/kg·m^{-2}	12
Parity/n (%)	12
Number of pregnancies/n (%)	0
Education level/n (%)	12
Exercise/n (%)	0
History of miscarriages/n (%)	0
Family history of diabetes/n (%)	6
History of diabetes/n (%)	0
Pregnancy complications (GDM)	0

Abbreviations: SFA = Saturated Fatty Acids; MUFA = Monounsaturated Fatty Acids; PUFA = Polyunsaturated Fatty Acids; Cr = Serum Creatinine; WBC = White Blood Cell; NLR = Neutrophil Lymphocyte Ratio; RBC = Red Blood Cell; HB = Hemoglobin; HCT = Hematocrit; PLT = Platelet; CRP = C-reactive Protein; TC = Total Cholesterol; TG = Triglycerides; HDL = High-Density Lipoprotein Cholesterol; LDL = Low-Density Lipoprotein Cholesterol; GDM = Gestational Diabetes Mellitus.

* Excluded due to excessive missing values

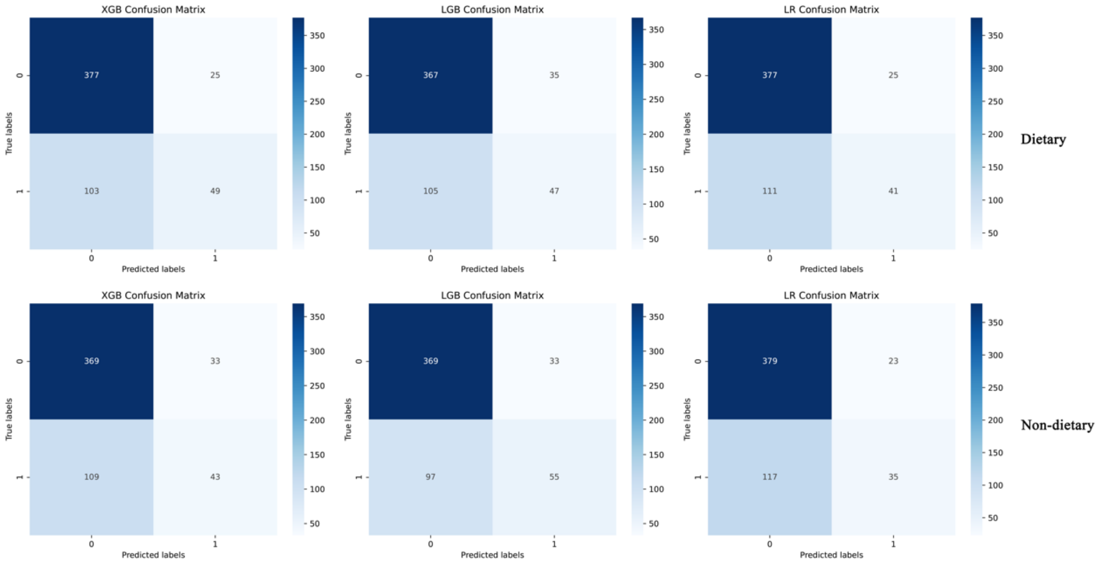

eFigure1. The confusion matrixes of the three GDM models

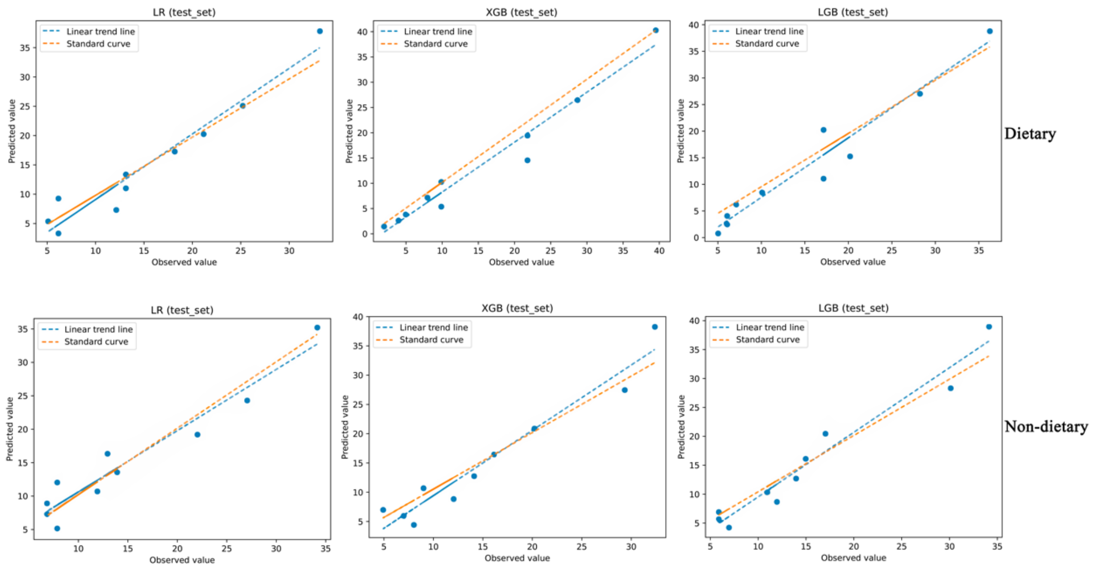

eFigure 2. The calibration curve for the test set of the three GDM models

Statistical methods: Independent Samples t-Test and Mann-Whitney U Test.

eTable 2. Comparison of Dietary Data and Clinical Data Between the Control Group and the GDM Group

Characteristics	Control (402)	GDM (152)	P value
Energy (Kcal)	1569.81±901.51	1543.33±608.68	0.626
Protein (g)	63.33±21.37	62.98±20.99	0.979
Fats (g)	43.10±21.93	47.39±29.72	0.166
Carbohydrates (g)	237.50±79.42	230.42±85.23	0.142
Dietary Fiber (g)	19.11±7.60	19.00±7.63	0.823
Calcium (mg)	744.77±322.54	749.67±313.68	0.832
Iron (mg)	17.62±6.95	16.98±6.35	0.339
Zinc (mg)	9.44±3.63	9.45±3.83	0.995
Selenium (μg)	43.40±18.84	45.84±24.79	0.494
Copper (mg)	2.58±1.87	2.68±1.85	0.590
Manganese (mg)	4.75±2.04	4.74±2.13	0.776
Magnesium (mg)	346.61±130.44	348.05±138.61	0.726
Sodium (mg)	804.56±429.15	796.06±398.70	0.912
Potassium (mg)	2683.11±1018.60	2648.53±1089.88	0.381
Phosphorus (mg)	1053.18±366.99	1071.05±396.90	0.760
Retinol (μgRE)	1293.51±715.54	1237.25±683.78	0.440
Vitamin E (mg)	19.23±8.90	19.42±9.06	0.920
Vitamin B1 (mg)	0.91±0.40	0.88±0.33	0.574
Vitamin B2 (mg)	1.17±0.50	1.16±0.45	0.911
Ascorbic acid (mg)	265.89±140.62	261.97±136.28	0.772
Niacin (mg)	12.75±5.72	12.76±6.65	0.609
Cholesterol (mg)	390.84±240.21	401.26±247.66	0.930
Water (g)	1378.81±525.98	1387.00±586.71	0.596
Iodine (μg)	40.03±31.92	36.99±18.64	0.968
Vitamin E (α-E) (mg)	5.61±2.79	5.77±2.66	0.296
Vitamin E ((β+γ)-E) (mg)	8.20±5.61	8.38±5.27	0.685
Vitamin E (δ-E) (mg)	3.39±1.87	3.38±2.35	0.174
Grain (g)	198.76±94.26	182.27±85.97	0.051
Legumes (g)	81.18±87.04	71.74±73.33	0.380
Vegetables (g)	648.88±390.32	628.54±418.93	0.260
Fungus and Algae (g)	29.78±32.91	25.91±31.36	0.253
Fruits (g)	578.38±332.82	590.49±429.43	0.479
Poultry (g)	12.62±20.83	12.00±21.81	0.149
Livestock Meat (g)	33.48±36.74	41.75±62.71	0.431
Dairy (g)	251.62±258.14	255.46±164.77	0.409
Eggs (g)	58.30±43.07	57.90±41.72	0.586
Aquatic Product (g)	48.51±51.88	55.70±65.06	0.322
Energy Composition Ratio (Carbohydrates) (%)	58.95±8.23	56.55±9.18	0.015
Energy Composition Ratio (Fats) (%)	24.95±7.21	26.95±7.23	0.007

Energy Composition Ratio (Protein) (%)	16.07±2.67	16.11±2.56	0.957
Energy from carbohydrates (Kcal)	898.19±304.36	868.91±328.64	0.108
Energy from fats (Kcal)	387.99±197.47	426.49±267.45	0.168
Energy from protein (Kcal)	245.65±85.25	246.18±96.76	0.809
high-quality protein	30.29±15.53	31.11±17.44	0.620
composition ratio of high-quality protein	48.05±13.29	49.09±12.18	0.400
low-quality protein	31.14±11.78	30.45±11.85	0.496
composition ratio of low-quality protein	51.84±13.24	50.91±12.18	0.451
animal-derived fats	22.41±14.74	25.49±23.84	0.368
composition ratio of animal-derived fats	52.60±17.87	52.32±16.66	0.868
plant-derived fats	20.70±14.38	21.91±12.01	0.035
composition ratio of plant-derived fats	47.40±17.87	47.68±16.66	0.868
SFA composition ratio	38.02±12.90	36.97±11.75	0.383
MUFA composition ratio	32.04±15.27	32.53±13.96	0.507
PUFA composition ratio	29.97±5.75	30.49±6.23	0.473
Cr (μmol/L)	35.96±6.90	40.34±28.65	0.288
Blood Sugar (mmol/L)	4.47±0.47	4.76±0.53	<0.001
WBC (10^9/L)	8.90±2.34	9.25±2.44	0.195
NLR	3.90±1.73	4.09±1.77	0.177
RBC (10^9/L)	4.01±0.46	4.09±0.46	0.113
HB (g/L)	121.22±12.15	123.74±11.93	0.034
HCT (%)	35.98±3.38	36.80±9.01	0.058
PLT (10^9/L)	222.60±55.97	226.06±59.20	0.524
TC (mmol/L)	5.61±1.00	5.41±1.09	0.026
TG (mmol/L)	2.46±0.99	2.74±1.43	0.071
HDL (mmol/L)	1.95±0.37	1.79±0.37	<0.001
LDL (mmol/L)	3.29±1.09	3.25±1.21	0.218

Abbreviations: GDM = Gestational Diabetes Mellitus; SFA = Saturated Fatty Acids; MUFA = Monounsaturated Fatty Acids; PUFA = Polyunsaturated Fatty Acids; Cr = Serum Creatinine; WBC = White Blood Cell; NLR = Neutrophil Lymphocyte Ratio; RBC = Red Blood Cell; HB = Hemoglobin; HCT = Hematocrit; PLT = Platelet; TC = Total Cholesterol; TG = Triglycerides; HDL = High-Density Lipoprotein Cholesterol; LDL = Low-Density Lipoprotein Cholesterol.
